# Atmospheric Pressure Plasma Deposition of Organosilicon Thin Films by Direct Current and Radio-frequency Plasma Jets

**DOI:** 10.3390/ma13061296

**Published:** 2020-03-13

**Authors:** Iryna Kuchakova, Maria Daniela Ionita, Eusebiu-Rosini Ionita, Andrada Lazea-Stoyanova, Simona Brajnicov, Bogdana Mitu, Gheorghe Dinescu, Mike De Vrieze, Uroš Cvelbar, Andrea Zille, Christophe Leys, Anton Yu Nikiforov

**Affiliations:** 1 Department of Applied Physics, Ghent University, Sint-Pietersnieuwstraat 41 B4, 9000 Gent, Belgium; Christophe.Leys@UGent.be (C.L.); anton.nikiforov@ugent.be (A.Y.N.); 2 National Institute for Lasers, Plasma and Radiation Physics, Magurele-Bucharest, MG-36, RO 077125 Ilfov, Romania; daniela.ionita@infim.ro (M.D.I.); andrada@infim.ro (A.L.-S.); brajnicov.simona@inflpr.ro (S.B.); mitub@infim.ro (B.M.); dinescug@infim.ro (G.D.); 3 Centexbel, Technologiepark-Zwijnaarde 70, 9052 Gent, Belgium; mdv@centexbel.be; 4 Department of Surface Engineering and Optoelectronics, Jožef Stefan Institute, Jamova cesta 39, 1000 Ljubljana, Slovenia; uros.cvelbar@ijs.si; 5 2C2T-Centro de Ciência e Tecnologia Têxtil, Universidade do Minho, Campus de Azurém, 4800-058 Guimarães, Portugal; azille@2c2t.uminho.p

**Keywords:** plasma deposition, atmospheric pressure plasma, thin film, organosilicon films, organosilicon precursors

## Abstract

Thin film deposition with atmospheric pressure plasmas is highly interesting for industrial demands and scientific interests in the field of biomaterials. However, the engineering of high-quality films by high-pressure plasmas with precise control over morphology and surface chemistry still poses a challenge. The two types of atmospheric-pressure plasma depositions of organosilicon films by the direct and indirect injection of hexamethyldisiloxane (HMDSO) precursor into a plasma region were chosen and compared in terms of the films chemical composition and morphology to address this. Although different methods of plasma excitation were used, the deposition of inorganic films with above 98% of SiO_2_ content was achieved for both cases. The chemical structure of the films was insignificantly dependent on the substrate type. The deposition in the afterglow of the DC discharge resulted in a soft film with high roughness, whereas RF plasma deposition led to a smoother film. In the case of the RF plasma deposition on polymeric materials resulted in films with delamination and cracks formation. Lastly, despite some material limitations, both deposition methods demonstrated significant potential for SiO_x_ thin-films preparation for a variety of bio-related substrates, including glass, ceramics, metals, and polymers.

## 1. Introduction

In recent years, the engineering of new materials with high biocompatibility has attracted interest from the industry, medical, and health sectors. The reason for this is a paradigm shift in biomedical engineering from modifying the physical, chemical, and mechanical properties of bulk material to re-engineering only the surface of the materials. One of the foremost strategies is implementing a thin-film layer deposition on the top surface of materials, such as non-woven fabrics, metals, or polymers, without affecting the bulk. Such surface modifications preserve the original material properties, since only a thin layer of about 10–500 nm is deposited.

Plasma treatment is established as an effective technique for surface functional modifications. Among the most used films for surface material modification are those that are based on inert silicon dioxide. Hexamethyldisiloxane (HMDSO), a monomer that cannot be polymerized following the conventional polymerization methodologies in a liquid phase. However, it can be polymerized during plasma treatments, by rearranging the radicals produced by its dissociation, is the most commonly used precursor for plasma deposition of these films [1]. SiO_2_-like films that were deposited by plasma polymerization of HMDSO/O_2_ mixtures were extensively studied in the last years for various applications, including the protection of metal and polymers, optoelectronics and optics [2], barrier films for food packaging, etc. In many reported cases, the performance of the plasma-deposited film was influenced by both its chemical composition and morphology.

One of the industry-interesting deposition methods is the atmospheric-pressure plasma deposition (APPD), which does not need expensive vacuum systems [3]. The APPD is expected to realize a low-cost and high-throughput processing with open-air systems [4]. Atmospheric plasmas still cannot fully replace low-pressure plasmas, especially when high-quality and film density with controlled chemistry are required, despite these promising potentials [5], although systematic studies on the effect of plasma excitation method on the polymerization process are lacking in the literature.

In this paper, two different APPD systems with RF and DC discharges that were used for the deposition of organosilicon films were evaluated. A novelty in this study is mainly related to the fact that different plasma generation methods produce films with the similar structure, and also a different point of precursor injection: in DC plasma jet, the precursor is directly introduced into plasma, whereas in RF plasma jet, the precursor is indirectly introduced in the afterglow region. To evaluate different substrate materials and their potential for APPD film, the thin organosilicon films are deposited on: glass, Teflon (polytetrafluoroethylene (PTFE)), polyetheretherketone (PEEK), stainless steel, titanium, and ceramics with HMDSO precursor. The rationality in our choice of substrate materials is related to their wide use in manufacturing biomaterials. As an example, PEEK is well known as a material for implants and PEEK based implants are considered to be an alternative to titanium- and ceramic-based implants in cranial, maxilla-facial, orthopedic, and spine surgeries [6]. Furthermore, stainless steel is one of the most used materials in medical devices, where good corrosion resistance and moderate strength are required [7,8]. Titanium applications include hip and knee prostheses, trauma/fixation devices, instruments and dental implants [9]. The deposition of the organosilicon films on these substrates is highly important, since they can improve biocompatibility. Moreover, these films can also be used as a barrier layer or composite matrix for drugs loading [10,11,12]. For these reasons, the required control of films chemistry and morphology is studied in this paper and its connection with different plasma sources with direct and indirect precursor injection.

## 2. Experimental Part 

The deposition of organosilicon films by using HMDSO as a gaseous precursor by an atmospheric pressure nitrogen DC and argon RF plasma jets was performed while using glass, ceramics, stainless steel, titanium, and polymeric materials polytetrafluoroethylene (PTFE) and polyetheretherketone (PEEK). It should be outlined here that, in both cases, the substrate was placed downstream regarding the plasma generation region. However, in the DC plasma jet, the samples were located in the afterglow region, where no plasma was present, whereas in the case of RF plasma jet deposition, the samples were exposed closed to nozzle exit and they were in direct contact with the expanding plasma. Both experiments have been done in scanning mode to perform homogenous film deposition.

### 2.1. DC Plasma Jet

**Figure 1:**
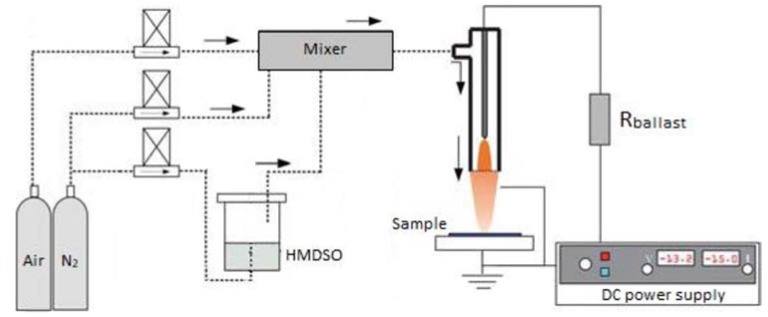
Scheme representing the atmospheric pressure direct current (DC) plasma deposition system.

Figure 1 presents the schematic diagram of direct current (DC) plasma jet, while details regarding the operation are listed elsewhere [13,14,15].

The DC plasma jet has a stable voltage and current without oscillations at a current that is higher than 10 mA, the plasma behaves as a glow discharge, as described in the previous study [16]. The current is 18.4 mA and the voltage is 18.0 kV. The DC jet is characterized by low heat transfer from the plasma source to allow for the substrate processing temperatures below 50 °C and it is therefore suitable for temperature sensitive substrates.

The substrate was positioned 10 mm away from the jet in the afterglow region. This was done to avoid thermal damage to the substrate. The DC jet was generated in N_2_ with a flow of 7000 sccm with an admixing of fixed airflow of 100 sccm and HMDSO (Sigma–Aldrich, St. Louis, MS, USA) with the recalculated precursor flow of 1 sccm (condition A) or 5 sccm (condition B). The variable ratio of HMDSO/O_2_ was used as a parameter to control the SiO_x_C_y_H_z_ film’s composition and morphology.

### 2.2. RF Plasma Jet

**Figure 2:**
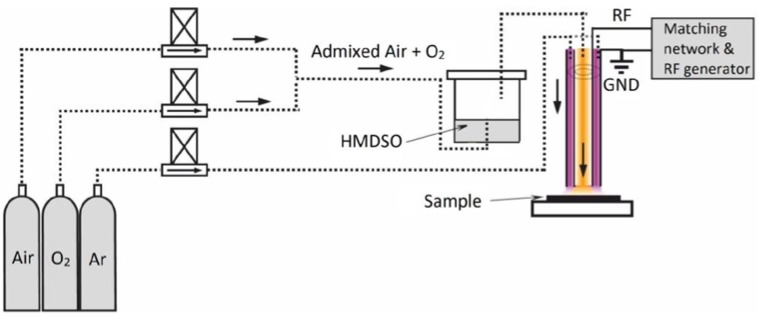
The schematic of the atmospheric pressure RF plasma deposition system.

The atmospheric pressure RF plasma jet that us presented in Figure 2 had a discharge configuration with bare electrodes (DBE) and is described in detail elsewhere [17,18]. The discharge needed no ballast and provides opportunities for an easy upscale for any required demand.

The RF and ground electrodes are represented by cylindrical tubes, mounted in a coaxial configuration allowing for the discharge to be ignited in the space that is delimited by the external cylindrical surface of the RF electrode and the inner cylindrical surface of the ground electrode (Figure 2, pink region). The annular gap between electrodes was set to 1 mm to ensure stable plasma operation.

Argon is used at a flow rate of 2500 sccm, which ensures the expansion of the plasma from the outlet nozzle of the jet, which creates an annular fingerprint of plasma on the substrate. The deposition process is carried out by the injection of HMDSO vapours admixed with oxygen (22 sccm), or adding also air (100 sccm) through the central tubular electrode, directly in the afterglow region. By this approach, the amount of precursor that was available for deposition was tuned from 1.7 sccm (condition A) and 7 sccm (condition B), similar to the conditions used with DC plasmas jet. The utilization of air ensured a similarity of approach considered for deposition with the DC plasma source. It should be noted that the distance between the nozzle and a substrate was set to 1 mm and the plasma was in contact with the substrate surface.

An overview of the operational parameters used for both DC and RF jet deposition is given in Table A1 (Appendix A).

### 2.3. Surface Characterization

The chemical surface composition was analyzed by X-ray photoelectron spectroscopy (XPS, PHI, City of Industry, CA, USA). XPS spectra were recorded with a PHI Versaprobe II spectrometer (PHI, City of Industry, CA, USA) employing a monochromatic Al K_α_ X-ray source operating at 100 W. All of the XPS measurements were performed in five points that were located in different parts of the sample. XPS is only measuring composition over 5–10 nm top lager and does not affect the film thickness (if thickness larger than 10 nm).

The survey spectra were recorded to obtain the elemental composition, while high resolution measurements were carried out to obtain further insight into the chemical bonds present in the plasma polymerized HMDSO films. The binding energies were all referred and corrected to the peak C1s at 285.0 eV. The HMDSO deposit morphology was additionally investigated by the scanning electron microscopy (SEM, FEI, Hillsboro, OR, USA) FEI S Inspect Scanning Electron. The samples were also analyzed by atomic force microscopy (AFM, Park Systems, Suwon-Si, Gyeonggi-Do, Korea) in order to obtain surface topography and roughness. The AFM measurements were performed with Park Systems, XE-100 microscope (Park Systems, Suwon-Si, Gyeonggi-Do, Korea) using a maximum scan area of 50 × 50 μm^2^, and a maximum vertical investigation height of 12 μm in non-contact mode.

The deposition rate of the films has been measured in previous published results [9]. For RF plasma jet, the deposition rate of the HMDSO layer on the surface 12.5 mm^2^ is 10 nm per 1 s. The thickness was measured in DC plasma in the range 172–444 nm min^−1^, depending on the operational conditions. All of the measurements were performed on Si wafer due to the complicated process of measuring on polymer and other substrates used here.

### 2.4. Discharge Characteristics and Deposition Temperature

**Figure 3:**
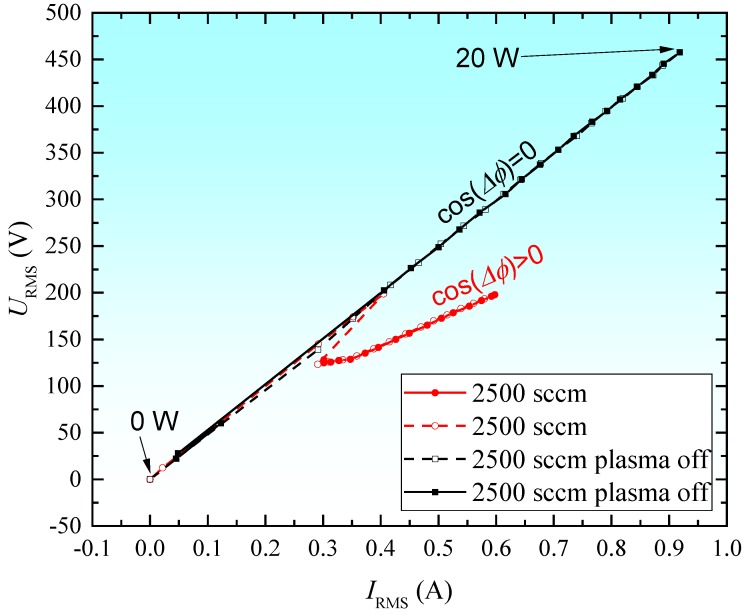
I-V characteristic for discharge configuration with bare electrodes (DBE) discharge (0–20 W, Ar flow 2500 sccm).

Figure 3 shows RF plasma V/I characteristics. The red curves are obtained in the presence of discharge and the black curves in its absence. In the absence of discharge, the current passes through the capacitor represented by the electrodes of the plasma source and the space between them, filled by the gas flow (argon). However, the active power is zero watts, because, in a purely capacitive circuit, the alternating voltage lags the current by 90 degrees (Δ*Φ* = −90°, cos(Δ*Φ*) = 0). The full points are obtained by incrementing the RF power value and the empty ones when decreasing it.

While considering, as a first approximation, the equivalent discharge circuit as a series RX circuit, where X represents the equivalent total reactance, and R represents the resistance of the plasma, the mean active power can be determined from the harmonic components with Equation (1):

(1) $$P_{AVR}=\overset{m}{\underset{n=1}{\sum }}{\left[U_{RMS}I_{RMS}\cos\Delta \phi \right]}_{n}$$

where P_AVR_ is the mean active power, U_RMS_ is the mean square value of the voltage, I_RMS_ is the quadratic mean value of the current, ΔΦ is the phase difference between the waveform of the current and the voltage, n is the order of the harmonic, and m is the maximum order of the harmonics, m = 6 in this case.

To extract harmonic, amplitude, and phase components, waveforms of current and voltage (I, V), after being acquired in the computer, were decomposed by a Fast Fourier Transform (FFT) algorithm. Applying Equation (1) of the harmonic components, the power injected into the discharge was determined. The results evidence that only about ¾ of the power input from the generator P_FWD_ effectively deposited in the discharge, for P_FWD_ = 15 W, we have P_AVR_ = 11.4 W in the presence of the discharge. The ¾ ratio is not significantly influenced by the gas flow.

The temperature of the gas phase in the afterglow of the DC jet was studied in previous work [16]. It should be outlined here that, in both cases, the substrate was placed downstream concerning the plasma generation region, as detailed in previous work [18]. It was shown the T_g_ at 1 cm distance from the discharge is below 56 °C. The RF plasma gas temperature was measured with a thermocouple and values in the range of 40–80 °C were obtained, depending on the applied RF power value and the gas flow rate. Under typical working conditions, the jet temperature is 43 °C, suitable for the treatment of temperature sensitive polymeric surfaces, such as PET (either as foil or fabric). Substrate temperatures have been made to measure in both cases. For DC and RF (N), the plasma substrate temperature was below 33 C and 62 C that was monitored with an IR camera (FLUKE, NL-5602 BD, Eindhoven, The Netherlands).

## 3. Results and Discussion

The chemical composition of the deposited films is identified and typologiesed in order to link plasma properties and its discharge parameters. Furthermore, the film quality is also analysed through the AFM and SEM techniques, to interconnect the discharge parameters with obtained film surface and morphology.

### 3.1. Chemical Composition

**Figure 4:**
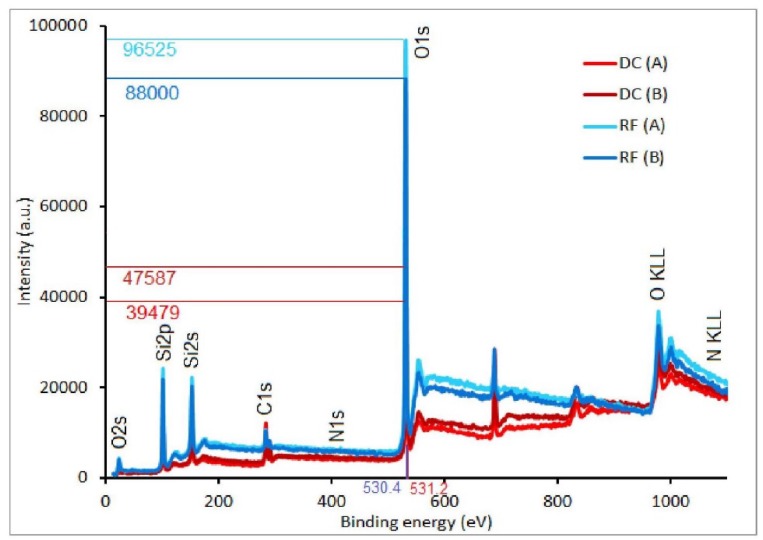
X-ray photoelectron spectroscopy (XPS) survey spectra of deposited films onto polytetrafluoroethylene (PTFE) substrates deposited by DC and RF plasma jets in conditions A and B.

The method of deposition, as well as a ratio of HMDSO/O_2_, can result in substantial variation of the film’s chemical composition [4]. With regards to the possible application of the organosilicon films it is well accepted that higher SiO_x_ content leads to fragile but harder films with low porosity which can be of interest e.g., in engineering barrier coatings [19]. To gain insights into the chemical composition of the deposits the XPS method has been applied in this work. It has to be emphasized that the penetration depth of about 5 nm limits the method and so only the top surface of the coatings is acceptable to this analytical methodology. Figure 4 presents the typical XPS survey spectra of the deposited films on the PTFE substrate prepared with the use of DC and RF plasma jet. As expected, the survey spectra indicate that the plasma deposited films are mainly formed by O, C, and Si, and along with an insignificant amount of N (below 1 at.%). The peak of F is appearing due to contamination in the XPS chamber from previous analysis, and so has been excluded from the analysis and calculation of the at. %. The elementary composition of the films is presented in Table A2 (Appendix A) for all coatings deposited with both the DC and RF plasma jet, respectively.

The absence of any substantial N-groups incorporation in DC plasma jet operating in pure N_2_ gas well agrees with the detailed study of DC plasma jet spectral properties that were performed in [20]. It is important to indicate that the XPS results show that the films obtained by DC and RF plasma jets have similar chemical composition, despite the different methods of deposition on various substrates and with different modes of the precursor injection. The amount of O in the deposits varies in the range between 54 to 62 at.% for DC plasma jet, and only slightly higher up to 57 to 66 at.% for RF plasma jet deposition. The Si content is also slightly higher for RF plasma deposits and is in range between 26 to 29 at.%. It is revealed that C content presented in deposits prepared by DC plasma jet reached a relatively low value of 22 at.% (PTFE substrate) in comparing with O, and even lower of only 14 at.% in the case of RF plasma. This clearly indicates strong precursor fragmentation and oxidation in both discharges. This observation is also in agreement with available literature. The observed formation of a low C-group contained films is, in our opinion, related to the fact that, in all experiments, the HMDSO: O_2_ ratio was very low with an excess of O_2_. The High O_2_ content in the gas phase and its intensive dissociation in conditions realized in both RF and DC plasma should result in the deposition of inorganic coatings that is confirmed by the XPS method.

**Figure 5:**
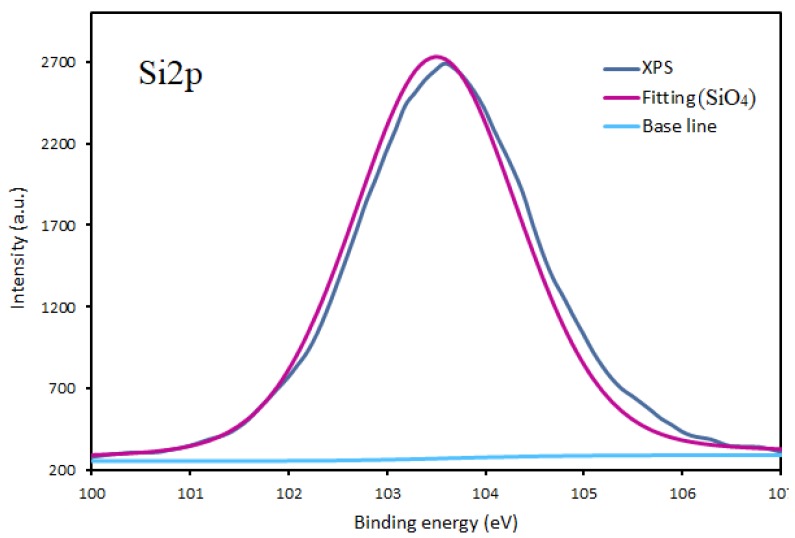
Fitting of Si2p peak of the thin film deposited onto titanium substrates by DC plasma jet in condition A.

It is expected that in conditions of atmospheric pressure plasma on the surface a carbon at hybridization state sp^2^ and Si-O-C group will be presented in large quantities. To gain a more detailed analysis of the functional groups on the top surface of the coatings, the detailed XPS spectra of Si2p, O1s, and C1s were measured. High-resolution XPS spectra in the Si2p region (Figure 5) were used to characterize the quality of the organosilicon films in terms of inorganic content.

The interpretation of the Si peak was done while considering four components, corresponding to (CH_3_)_3_SiO, (CH_3_)_2_SiO_2_, CH_3_SiO_3_, and SiO_4_ bonds located at energies 101.5 ± 0.1 eV, 102.1 ± 0.1 eV, 102.8 ± 0.1 eV, and 103.4 ± 0.1 eV, respectively [21,22]. No other possible bonds were considered in the fitting procedure, including SiO_4_^+^ 2p1/2, due to the negligible contribution to the fitting results. During the fitting procedure, the full width at half maximum (FWHM) for all of the Si2p components was kept in the range from 1.3 to 1.9 eV. Fitting shows deconvolution of the high resolution spectrum of the Si2p region and the associated fitted peak of plasma-polymerized HMDSO films that were obtained in nitrogen/air mixtures in DC plasma jet afterglow on Ti substrate. The contribution of SiO_x_ bond located at an energy of 103.4 ± 0.1 eV to the Si 2p peak of the films deposited by using DC plasma jet is dominated, regardless of the precursor flow or the substrate type. Very similar results with the predominance of SiO_x_ bonds were also obtained for A, B conditions in both RF and DC plasmas in all conditions on all substrates (not presented here). The fitting of the Si2p peak (Figure 5) indicates that the films that were deposited on different substrates consist of Si-O-Si bond types with the tetrahedron structure of SiO_4_. In the case of the RF plasma jet, the same trends are detected with the Si2p peak mainly composed (95%) of SiO_x_ (103.4 ± 0.1 eV) bond, while the other three peaks that were assigned to (CH3)_n_SiO_4−n_ type of bonds have none or little contribution. This is in good agreement with [22], where the oxygen content of the film was found to increase, with a corresponding decrease in carbon concentration as a result of the increased level of plasma exposure of the films during deposition. The obtained results indicate that, despite the difference in the plasma excitation methods and the point of precursor injection during deposition, both methods provide almost complete oxidation of the precursor, leading to the formation of SiO_x_ film on any substrate studied in this paper. In HMDSO-containing plasma, the molecules are fragmented by the dissociation of methyl groups and, at the same time, the stronger Si–O bindings are preserved. Similarly, A. Sonnenfeld et al. [22] has found that the admixing of O_2_ in nitrogen plasma gas leads to the dominant role of atomic O in the mechanism of HMDSO polymerization and it can provide a way to control the ratio between silicon and oxygen in the SiO*_x_* film from polymer-like (1:1) to quartz-like (1:2) structures.

**Figure 6:**
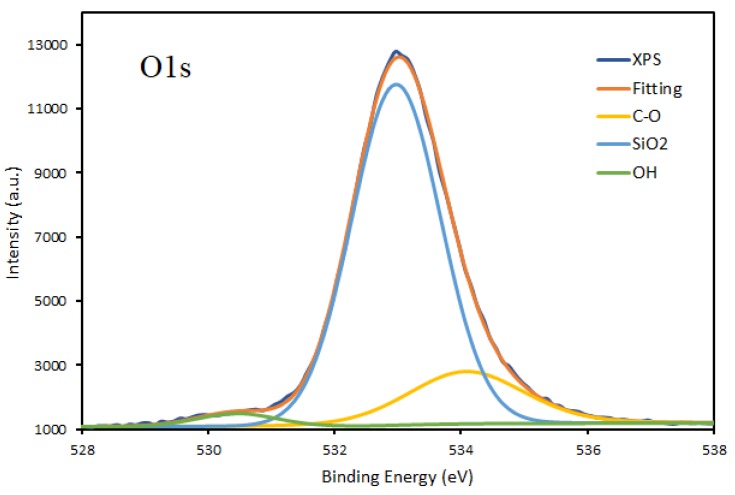
Fitting of O1s peak of the thin film deposited onto titanium substrates by DC plasma jet in condition A.

**Figure 7:**
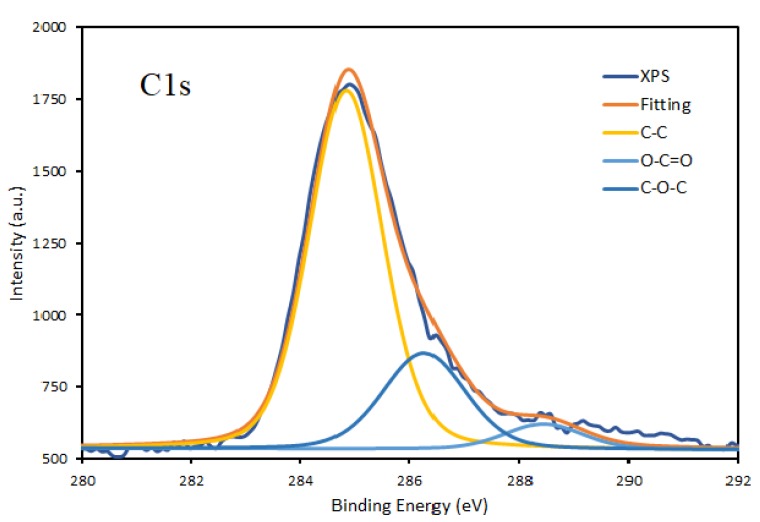
Fitting of C1s peak of the thin film deposited onto titanium substrates by DC plasma jet in condition A.

Interestingly, a detailed analysis of Si2p peak indicates very low C content in the film, which seems contradictory with the elemental composition of the coatings obtained based on survey XPS spectra. Because of the discrepancy of results fitting for high resolution spectra of both C1s and O1s for DC plasma deposition were performed and presented in Figure 6 and Figure 7.

**Figure 8:**
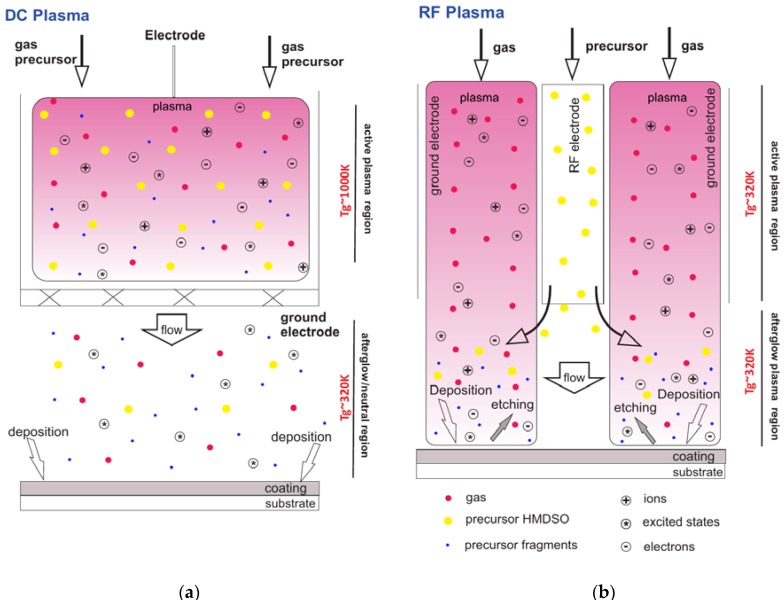
Schematic representation of the deposition process in DC (**a**) and RF (**b**) plasma jets.

The fitting of the O1s peak clearly shows the dominant Si-O bond in the deposits. The fitting also indicates the presence of the C-O bond and very low OH bond, probably appearing due to water uptake during the storage of the samples before XPS analysis. The results of O1s peak analysis fit well with the deconvolution of Si2p spectra and indicates an almost negligible content of C-Si-O bonds. The C1s peak has a relatively complicated profile in all of the samples obtained by both DC and RF plasma deposition, as presented in Figure 8. The peak has been deconvoluted into three contributions corresponding to C-C, O-C=O, and C-O-C bonds. It has to emphasize that no contribution of C-Si around 282.2 eV [23,24,25] was detected.

This observation corresponds to a conclusion that the obtained deposits consist of two fractions, namely almost inorganic SiO_4_ film with inclusions of carbon containing groups, but the C groups are not bonded with Si. The precursor of HMDSO is almost completely dissociated under discharge action and the C groups are co-deposited on the surface. Probably, partially high C content detected on the top surface of films by the XPS method can be explained by the unavoidable contamination of the samples during a storage time A difference in the film’s composition and C at.% on deposits that were obtained in DC and RF plasma with a lower C content on RF plasma engineered surfaces, in our opinion is related to the difference in the injection point of precursor and its different residence time in the plasma zone, as indicated in Figure 8.

**Figure 9:**
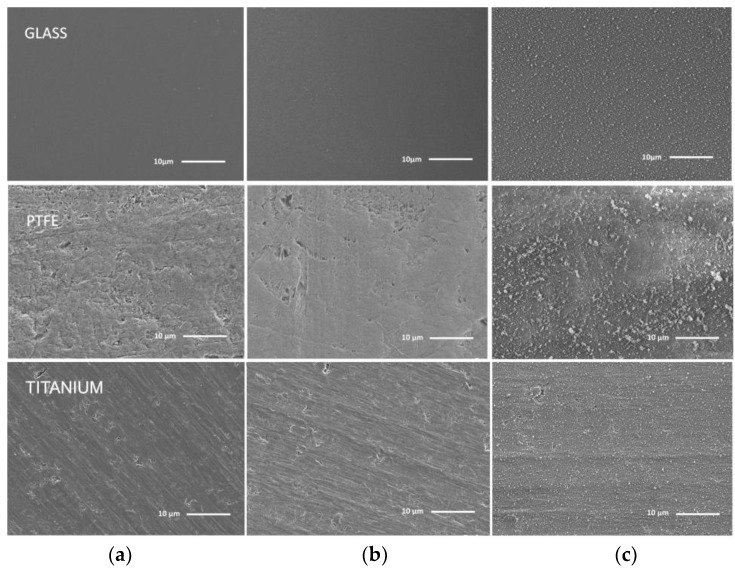
Scanning electron microscopy (SEM) images of thin films deposited on glass, PTFE and titanium in DС plasma jet (nitrogen/air): (**a**) Control, (**b**) Condition A, and (**c**) Condition B, respectively.

It was revealed by X. Zhu et al. [26] that the mechanisms of deposition could have a strong impact on the chemical composition of the films. Accordingly, the difference in composition between DC and RF plasma deposition is expected. Since, in DC plasma jet, there is no direct flux of active species, except N_2_ metastables, on the substrate the coated surface has a carbon content as high as 22 at.% C. This is due to the fact that C-containing radicals generated in gas phase are forming carbon-containing weak bonds on the substrate and incorporated on the film surface at high concentration. Whereas, in case of the films that were obtained in RF plasma (Figure 9), there is a substantial flux of various active species on the substrate surface that results in the etching of weak C- bonds from the surface, and, accordingly, Si and O content increase and C-bonds are partially removed, which is confirmed by the XPS results. Silica-like films are obtained as a result of RF plasma deposition with low C-contamination of the deposits.

### 3.2. Films Morphology and Topography

#### 3.2.1. Surface Morphology

**Figure 10:**
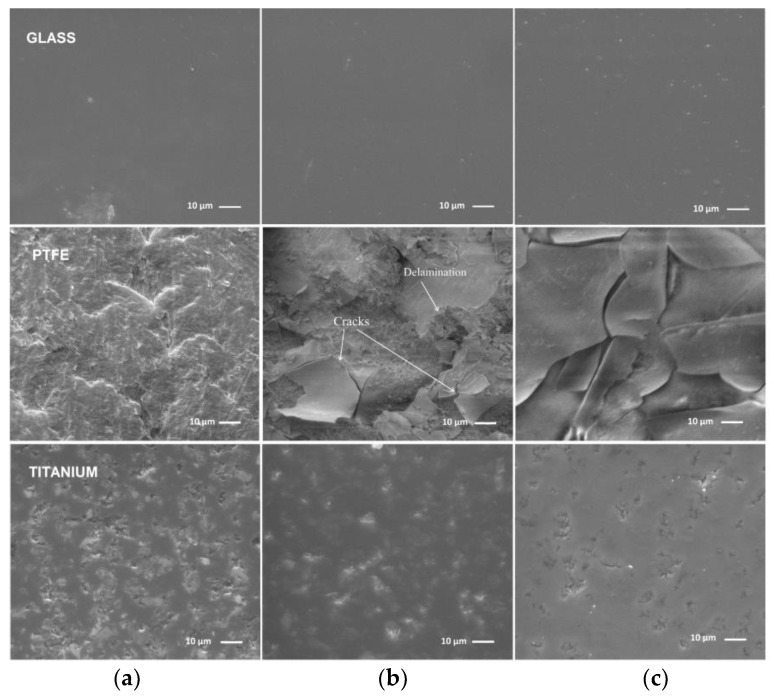
SEM images of the films on glass, PTFE and titanium deposited by the RF plasma jet: (**a**) Control, (**b**) Condition A, and (**c**) Condition B, respectively.

Surface morphology is expected to have a strong impact on the film suitability in final applications. The films deposited on various substrates (ceramics, glass, stainless steel, titanium, PEEK, and PTFE) were additionally investigated by SEM to study the effect of plasma source operational parameters on the surface morphology. Figure 9 and Figure 10 represent cases of morphology for DC and RF plasma deposits on the glass, PTFE, and titanium, being chosen as representative for glass, polymeric, and metallic types of substrates, respectively. In Figure 9b, the films were deposited in DC plasma afterglow at low content of HMDSO. On the other hand, Figure 9c shows that, at a high content of HMDSO, the films include a considerable amount of SiO_x_ nanoparticles, probably due to the nucleation of the precursor in a gas phase.

**Figure 11:**
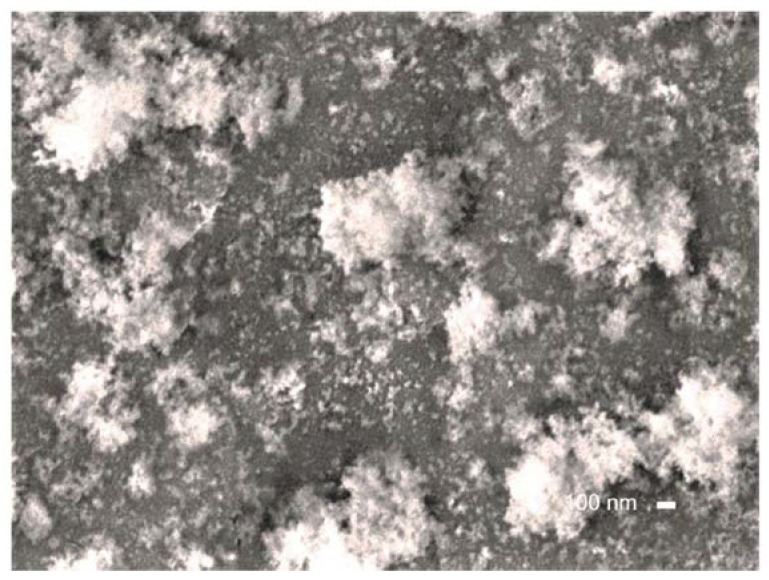
High resolution SEM image of the film on Ti substrate from Figure 9c in DC plasma jet.

High resolution SEM (Figure 11) reveals that, on Ti substrates, the size of the powder nanoparticles is about 200–600 nm. For the lower HMDSO flow, the film surface is smooth and continuous, no pinholes and nanograins are observed.

As the monomer flow rate is increased, a slight change of the surface features can be determined. Deposition on a polymer substrate, e.g., PTFE or PEEK, at condition B is characterized by the precursor excess and that results in a strong gas phase aggregation of the particles and formation of micro-clusters of 1–2.5 μm size. Twomey et al. also reported the effect of precursor flow rate [27], who observed that an increase in the precursor residence time within the plasma zone leads to the growth of particles with the sizes up to 300 nm. In general, this process has many analogies with the thermal chemical vapour deposition, where precursor concentration is the key parameter in the engineering of defect-free, high-quality films [28,29]. Previous investigations of organosilicon film engineering [30] indicated that the dust particles that are grown in the gas phase are the main source of the film’s contamination, which could cause severe defects [30]. The presence of the C-H and growth of high purity, uniform, and smooth SiO2-like layers was only achieved at low precursor flow corresponding to condition A [31].

Similarly, Figure 10 shows samples, which correspond to Figure 9, but that were deposited by RF plasma. It is determined that the organosilicon deposits are conformal to the substrate and the morphology of the surface does not significantly change during deposition to the glass and titanium. In contrast to the previous observation regarding the formation of particulates in DC plasma jet, the RF plasma deposits was not shown any particles on the surface, even at high HMDSO flow, Figure 10c. The precursor in the case of RF plasma is injected in the afterglow region, as displayed on Figure 8. Correspondingly, a residence time of HMDSO in the active plasma zone is rather short (about 60 μs), which is not enough to initiate the nucleation process in the gas phase. In the case of Ti, the defects on the surface get only partially covered upon the deposition process. At lower HMDSO flow, there are many imperfections on the surface, while, in the case of higher precursor flow, their number is diminished and a tendency of film nucleation along the defect borders is noticeable. Considerable different morphology is observed on both PEEK and PTFE polymer materials. For the PEEK material, which is rigid (no elastic deformation), cracks along the whole film surface are observed for lower HMDSO flow, while a smooth surface is attained with high HMDSO flow. The PTFE (soft and elastic material) surface is covered by a thin film, which has cracks and exfoliation. This observation is different from films that were obtained in DC plasma where no cracks are detected.

#### 3.2.2. Surface Topography 

**Figure 12:**
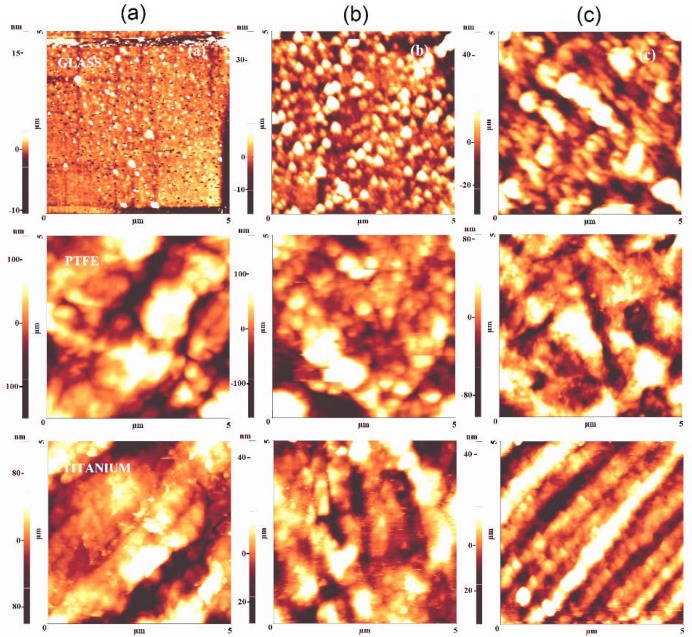
Atomic force microscopy (AFM) images of thin films deposited on glass, PTFE and titanium in DC plasma jet (nitrogen/air): (**a**) Control, (**b**) Condition A, and (**c**) Condition B.

**Figure 13:**
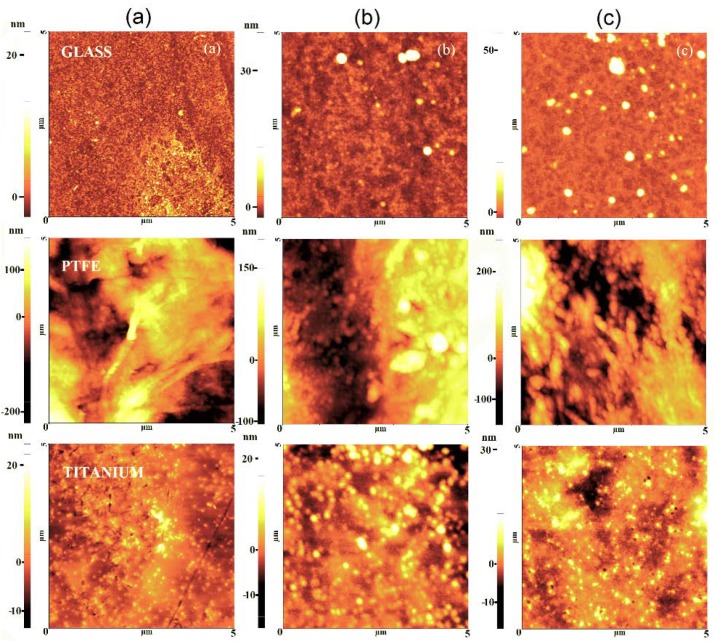
AFM images of thin films deposited on glass, PTFE and titanium in RF plasma: (**a**) Control, (**b**) Condition A, and (**c**) Condition B, respectively.

The AFM method in the non-contact mode was utilized to study the topography of the surfaces. Figure 12 and Figure 13 represent films deposited by DC plasma jet and RF plasma on glass, PTFE, and titanium substrates, respectively. The glass roughness RMS value, before the deposition, was varied between 0.8–1.8 nm. The deposited SiO_x_ films at low precursor flow rate slightly increase the roughness RMS value to 3.7 nm and 8 nm in the case of DC and RF plasma, respectively. The increase of the HMDSO flow rate leads to smoother films with RMS roughness of around 3-4 nm, as indicated in Figure 12 and Figure 13c. An overview of the surface morphology of the samples prepared by DC and RF methods of deposition is given in Table A3 (Appendix A).

Films that were obtained using both plasma sources exhibit similar behavior with smooth surfaces engineered at low precursor concentration and the formation of some nanoparticles at a high flow rate of precursor, especially in the case of DC plasma with a long plasma region residence time of gas. This observation is in agreement with the morphology results that were obtained by SEM.

The evolution of surface topography during plasma polymerization on polymeric materials is represented on the example of PTFE that is seen in Figure 12 and Figure 13. The results confirm that in DC plasma jet, the initial substrate roughness of 50 nm decreased upon deposition at low precursor rate to 19.8 nm and up to 31 nm for the condition of high flow rate, while, for the RF plasma jet, practically no change in roughness is observed, due to conformal substrate coverage. Changes of the surface roughness in the case of DC plasma are explained by the agglomeration of HMDSO nanoparticles created in the plasma zone that is filling the micro cracks and holes on the substrate surface. Gil et al. observed a similar mechanism [32], where nanoparticles with a size of about 50 nm were detected. The proper adjustment of precursor flow rate and method of injection is of critical importance for engineering of the conformal films with very low RMS roughness, as observed in this work as well as in an earlier paper by Aresta et al. [33] capable to reach films of 1.5 nm of RMS roughness of film by plasma deposition.

## 4. Conclusions

Atmospheric pressure plasma jets that were sustained by DC and RF power were used for the deposition of organosilicon films onto various substrates. Two methods of precursor injection: a) directly in the RF plasma region and b) indirectly in the afterglow of DC plasma were tested and analyzed in terms of their influence on deposited film chemistry and morphology. It was demonstrated that, even in the case of high N_2_ concentration in the plasma forming gas, the films are mainly composed of O, Si, and C and characterized by a negligible amount (below 0.83 at.%) of nitrogen containing groups. The deposited films are characterized by low C content in the range 6–20 at.%, with the rest of the film being attributed to the formation of organosilicon coating with more than 90 at.% of SiO_4_ bonds.

This is associated with the efficient oxidation process of HMDSO, ensured by the introduction of air/oxygen gas into the plasma. It was also noticed that the type of precursor injection method has very little impact on the film’s chemical composition. However, it is possible to obtain a large variety of films with respect to the surface morphology, according to the plasma source type and method of precursor injection. The RF plasma jet deposition resulted in a film with good surface uniformity. The utilization of DC plasma jet results in rougher films, which dominate the substrate features, especially at high HMDSO flow. It was proved that deposition in the afterglow mode performed in DC plasma is strongly sensitive to the precursor flow rate. At a high HMDSO flow of 5 sccm, a considerable amount of nanoparticles was detected on the films due to nucleation in the gas phase.

## Appendix A

**Table A1:** Experimental conditions for DC and RF plasma jets.

Plasma Jet	DC	RF
Current	18.4 mA	−
Voltage	18.0 kV	−
Power	−	11.4 W
Gas Flow	N_2_–7000 sccm	Ar–2500 sccm
Gas Flow (HMDSO)	A–1 sccm, B–5 sccm	A–1.7 sccm, B–7 sccm
Distance to substrate	10 mm	1 mm
Spot size	10 mm	12.5 mm

**Table A2:** Atomic concentration of elements in the films deposited by DC plasma jet (1) and RF plasma jet (2) for various air flow rates and several substrate materials.

DC Plasma Jet						RF Plasma Jet				
Materials	Precursor, sccm	O (%)	C (%)	Si (%)	N (%)	Precursor, sccm	O (%)	C (%)	Si (%)	N (%)
Ceramics	1	61	16.2	22.7	0	1.7	64.8	6.3	28.2	0.6
	5	61.2	14.3	23.6	0.83	7	57.8	14.8	26.8	0.5
Stainless steel	1	58.7	17.3	23.5	0.47	1.7	65.4	7.4	27.2	0
	5	62.4	12.2	25.4	0	7	65.4	5.8	28.7	0
Titanium	1	62.4	12.9	24	0.76	1.7	63.7	7.7	28.1	0.5
	5	61.2	12.9	25.8	0.05	7	65.2	5.8	29	0
Glass	1	61.1	12	26.1	0.79	1.7	64.7	7.6	27.1	0.6
	5	61.9	11.5	25.8	0.77	7	65.1	6.5	28.3	0.2
PEEK	1	60	15.8	24.1	0	1.7	66.2	5.5	28.2	0.1
	5	62	11.6	25.9	0.47	7	65	6	29	0
PTFE	1	54.3	22.5	23.2	0	1.7	63.93	9.16	26.81	0
	5	55.4	20.6	23.2	0.72	7	62.3	11	26.7	0

**Table A3:** Comparison morphologies of deposited coatings for DC and RF plasma jets.

Plasma Jet	DC	RF
Current	18.4 mA	−
Voltage	18.0 kV	−
Power	−	11.4 W
Gas Flow	N_2_–7000 sccm	Ar–2500 sccm
Gas Flow (HMDSO)	A–1 sccm, B–5 sccm	A–1.7 sccm, B–7 sccm
Distance to substrate	10 mm	1 mm
Spot size	10 mm	12.5 mm
